# Anatomy of the nerves, vessels, and muscular compartments of the forearm, as revealed by high-resolution ultrasound. Part 1: overall structure and forearm compartments

**DOI:** 10.1590/0100-3984.2021.0030

**Published:** 2021

**Authors:** Áurea V. R. Mohana Borges, Sergio A. L Souza

**Affiliations:** 1 Department of Radiology, School of Medicine, Universidade Federal do Rio de Janeiro (UFRJ), Rio de Janeiro, RJ, Brazil.

**Keywords:** Ultrasonography/methods, Forearm/anatomy & histology, Radial nerve/anatomy & histology, Median nerve/anatomy & histology, Ulnar nerve/anatomy & histology

## Abstract

In recent decades, high-resolution ultrasound (HRUS) has revolutionized the morphological and structural evaluation of peripheral nerves and muscles, revealing details of the internal structure of the neural fascicles and muscle architecture. Applications range from diagnostics to interventional procedures. The anatomy of the forearm region is complex, with several muscles and an extensive network of vessels and nerves. To guarantee the success of the evaluation by HRUS, knowledge of the normal anatomy of the region is essential. The aim of these two companion articles is to present the normal anatomy of the nerves and compartments of the forearm, as revealed by HRUS, as well as the relationships between the main vessels and nerves of the region. Part 1 aims to review the overall structure of nerves, muscles and tendons, as seen on HRUS, and that of the forearm compartments. We present a practical approach, with general guidelines and tips on how best to perform the study. Part 2 is a pictorial essay about compartment vascularization and cutaneous innervation. Knowledge of the normal anatomy of the forearm improves the technical quality of the examinations, contributing to better diagnoses, as well as improving the performance and safety of interventional procedures.

## INTRODUCTION

In recent decades, high-resolution ultrasound (HRUS) has revolutionized the morphological and structural evaluation of peripheral nerves and muscles, revealing details of the internal structure of the neural fascicles and muscle architecture^**(1-6)**^. Its applications range from the field of diagnostics to that of interventional imaging, such as the guidance of therapeutic injections and nerve blocks for anesthesia. HRUS allows a better topographic localization of the neural lesion and can identify partial lesions, the location of which can be difficult to discern in a clinical analysis and by electroneuromyography^**(7)**^. Therefore, HRUS can optimize the selection of the ideal area for surgery. Such advances are added to the established advantages of the modality, such as dynamic evaluation, simultaneous correlation with clinical symptoms, and greater ease of contralateral comparison. To guarantee the success of the HRUS evaluation, knowledge of the normal anatomy of the region under study is essential. The forearm is a region of high complexity, with 20 muscles and an extensive network of vessels and nerves^**(8)**^. To facilitate understanding of its anatomy, the forearm is usually divided into fascial compartments^**(9,10)**^.

The aim of these two companion articles is to present the normal anatomy of the nerves and compartments of the forearm, as revealed by HRUS, as well as the relationships between the main vessels and nerves of the region. Part 1 aims to review the overall structure of nerves, muscles and tendons, as seen on HRUS, and that of the forearm compartments. We present a practical approach, with general guidelines and tips on how best to perform the study. Part 2 is a pictorial essay about compartment vascularization and cutaneous innervation. The articles are illustrated with images obtained with a high-resolution broadband 18-5 MHz linear transducer. All individuals depicted in the images were volunteers and gave written informed consent.

## TECHNICAL CONSIDERATIONS

Transducers that operate in the frequency range between 1 and 70 MHz are available in clinical practice, and new transducers have been introduced to the market more quickly than in previous decades^**(11,12)**^. The new high-frequency ultrasound (HFUS) and ultra-high-frequency ultrasound (UHFUS) transducers have provided significant improvements in image spatial resolution. However, their widespread use is limited by their low penetration capability^**(11,13)**^.

There are various types of nerves in the forearm, and the depth at which they are found will determine the choice of transducer to be used. The recommendation is to use an 18 to 20 MHz linear broadband transducer initially, for a simultaneous gain of penetration and resolution. In a thicker forearm, a transducer with a lower frequency range, such as 12 to 15 MHz, can be used in order to guarantee penetration. For the evaluation of cutaneous nerves or nerve segments in a very superficial position, the use of HFUS and UHFUS transducers may soon become routine as they become more widely available. Therefore, in daily clinical practice, it is common to use more than one transducer with different penetration ranges, with the aim of optimizing neuromuscular evaluation.

**Figure 1:**
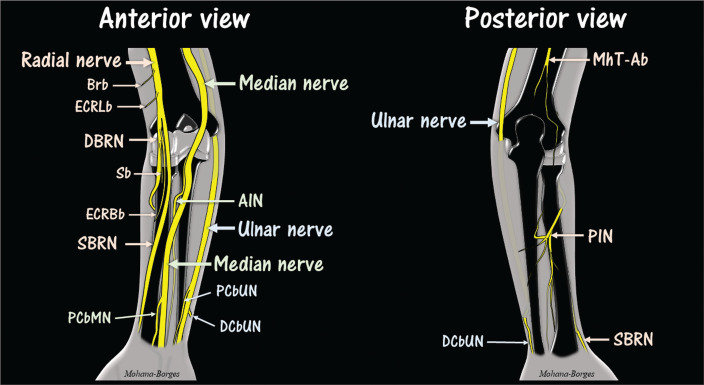
Diagrams of the major nerves in the distal arm, elbow, and forearm. Note the division of the radial nerve into its deep and superficial branches (DBRN and SBRN, respectively) at the level of the elbow. The radial nerve also has a brachioradialis muscle branch (Brb) and extensor carpi radialis longus muscle branch (ECRLb), illustrated in the anterior view diagram, as well as a medial head of the triceps and anconeus muscles branch (MhT-Ab), illustrated in the posterior view diagram. The MhT-Ab originates at a higher level in the arm (not shown). The DBRN gives off a branch to the supinator muscle (Sb) and may give off a branch to the extensor carpi radialis brevis muscle (ECRBb), as illustrated in the anterior view diagram. The ECRBb can originate directly from the radial nerve or from the SBRN. In the posterior view diagram, note the various branches of the posterior interosseous nerve (PIN). The PIN is the distal continuation of the DBRN. The median nerve gives off two main branches above the wrist: the anterior interosseous nerve (AIN) and the palmar cutaneous branch of the median nerve (PCbMN). The AIN arises below the elbow from the dorsolateral aspect of the median nerve and continues to a deep position in front of the interosseous membrane. The ulnar nerve gives off two main branches above the wrist: the palmar cutaneous branch of the ulnar nerve (PCbUN) and the dorsal cutaneous branch of the ulnar nerve (DCbUN). Median and ulnar nerve branches to the forearm muscles are not shown in the diagrams.

In general, the median nerve, the ulnar nerve, and the superficial branch of the radial nerve are easily visualized throughout their extent in the forearm^**(14)**^, as detailed in Table 1 and illustrated in Figure 1. The deep branch of the radial nerve is also observed in its entirety relatively easy, although its identification between the heads of the supinator muscle can be impaired in cases of muscle hypertrophy, with reduction of the perineural fatty plane^**(3,15)**^. Because of their deep location and small caliber, the interosseous nerves and their branches may be more difficult to visualize in practice (Table 1).

**Table 1:** Technical considerations regarding the nerves in the forearm.

Nerve Branch	Comments
Median	Easy to visualize—detection rate of 100% reported by Gruber et al.^(14)^; mean CSA = 6.98 mm^2(3)^.
Palmar cutaneous	Originates from lateral side of the median nerve in the distal 2/3 of the forearm with variable distance to the wrist; relatively easy to visualize—detection rates of 83% and 100% reported by Tagliafico et al.^(16)^ and Jeong et al.^(17)^, respectively; CSA = 0.5-0.7 mm^2^ and diameter = 0.8-1 mm in the Tagliafico et al. study^(16)^; mean diameter = 0.26 mm^2^ in the Jeong et al. study^(17)^.
Ulnar	Easy to visualize—detection rate of 100% reported by Gruber et al.^(14)^; mean CSA = 6.3 mm^2(3)^.
Palmar cutaneous	Originates from the lateral side of the ulnar nerve in the distal 1/3 of the forearm; relatively easy to visualize—detection rate of 78% reported by Kim et al.^(18)^; mean CSA = 0.3 ± 0.1 mm^2(18)^.
Dorsal cutaneous	Originates from the medial side of the ulnar nerve in the distal 1/3 of the forearm; proximal portion easy to visualize—detection rate of 100% reported by Kim et al.^(18)^; distal superficial segment, although visible, often difficult to differentiate from subcutaneous fat; mean CSA = 1.6 mm^2^ and mean diameter at origin = 2.4 mm^(19)^.
Radial Superficial	Easy to visualize—detection rates of 100% and 95% reported by Gruber et al.^(14)^ and Causeret et al.^(19)^, respectively; mean CSA = 1.97 mm^2(3)^.
Deep	Relatively easy to visualize; identification may be impaired in cases of supinator muscle hypertrophy with reduction of perineural fatty plane; CSA = 0.50-1.42 mm^2^ in Babaei-Ghazani et al.^(15)^ and mean CSA = 2.11 mm^2^ in Vlassakov et al.^(3)^.
Anterior interosseous	Visualized with some degree of difficulty due to its deep location and thinness, particularly in the distal 1/3 of the forearm and in cases of muscle hypertrophy; anterior interosseous vessels used as landmarks.
Posterior interosseous	Visualized with some degree of difficulty due to its deep location and thinness, especially in the distal 1/3 of the forearm and in cases of muscle hypertrophy; posterior interosseous vessels used as landmarks.
Lateral antebrachial cutaneous	Terminal sensory branch of the musculocutaneous nerve^(19)^; relatively easy to visualize in the elbow and proximal forearm; may be difficult to differentiate from the subcutaneous fat in the distal 2/3 of the forearm; cephalic vein used as a landmark because the nerve typically runs along the vein path; mean CSA = 3.3 mm^2^ and mean diameter = 1-2 mm^(19)^.
Medial antebrachial cutaneous	Direct branch of the brachial plexus that is visualized in the subcutaneous near the basilic vein; may be difficult to differentiate from the subcutaneous fat in the distal 2/3 of the forearm; approximate diameter = 1-2 mm^(20)^.
Posterior antebrachial cutaneous	Branch of the radial nerve, originating in the posterolateral arm; visualization is possible, although technically more difficult^(21)^; identified above the extensor digitorum communis muscle along the forearm (used as a landmark); may be difficult to differentiate from the subcutaneous fat; approximate diameter = 1-2 mm^(20)^.

CSA, cross-sectional area.

The cutaneous branches of the median and ulnar nerves, which originate in the forearm (Figure 1), are usually relatively easy to identify^**(16-19)**^, as explained in Table 1. The cutaneous nerves of the forearm are better visualized in the proximal portion of the forearm, where they are of a larger caliber^**(19-21)**^, as noted in Table 1. It can be difficult to differentiate those nerves from subcutaneous fat, particularly in the distal part of the forearm. Further studies are needed in order to establish the detection rate of these subcutaneous nerves in different forearm segments, in particular with the advent of new HFUS and UHFUS transducers.

## GENERAL STRUCTURE OF NERVES, MUSCLES, AND TENDONS ON HRUS

### Nerves

In HRUS, nerves are evaluated along their long and short axes^**(4)**^. Those neural axes do not always match the anatomical reference planes used in other imaging methods. The field of view is also smaller in ultrasound than in magnetic resonance imaging and computed tomography. Therefore, other references become more important in HRUS, such as the distance from the nerve to a joint or soft-tissue landmark; the level of emergence of a branch or division of the nerve; proximity to a particular muscle, vascular structure, or bone edge; and the point at which the nerve changes course.

The morphological and structural parameters of the nerves evaluated with HRUS are cross-sectional area, transverse diameters, changes in caliber, echogenicity pattern, and fascicle characteristics^**(4,13)**^. Nerves are malleable structures that change shape depending on the pressure and movement of muscles and tendons. Therefore, in HRUS, there is a preference to measure the area instead of the transverse diameters, except in cases of very small nerves, the area of which may be difficult to measure. In addition, HRUS allows dynamic and quantitative evaluations of nerves, areas of study that are still in development^**(22)**^.

### Internal nerve structure and the fascicular pattern

**Figure 2:**
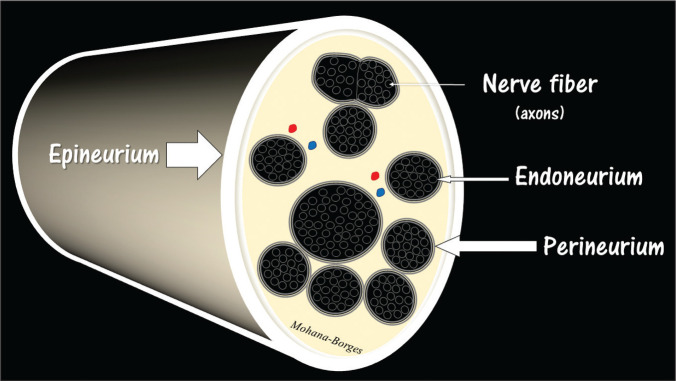
Simplified diagram of the composition of the peripheral nerve, showing a nerve fiber and how the layers of connective tissue and collagen are organized in the epineurium, perineurium, and endoneurium. The epineurium peripherally encompasses the nerve as a whole, the perineurium surrounds the nerve fascicles, and the endoneurium surrounds the individual nerve fibers.

A study conducted in the 1990s, using a 15 MHz transducer, elucidated the ultrasound aspects of peripheral nerves, correlating ultrasound images with histological findings^**(1)**^. As observed on HRUS, those nerves have an arrangement composed of hypoechoic and hyperechoic structures that resemble honeycombs. On histology, these structures correspond to the fascicles and epineurium, respectively (Figure 2). Because of their relative thinness, the perineurium and endoneurium have been undetectable by ultrasound, at least until now. It is possible that the new UHFUS transducers will change that perspective.

Conditions that result in changes in the usual fascicular pattern include edema, fibrosis, changes in the proportion of fat, and hypervascularization^**(13)**^. Those conditions, in turn, may be associated with several diseases^**(13)**^. In general, vessels are not detectable inside the normal nerve, because of their very small dimensions. When they are detected, it is usually because there has been an increase in their size caused by hyperemia, typically indicating a problem. The larger-caliber vessels are easily distinguished from nerves and other structures by the Doppler technique^**(23)**^.

### General guidelines for HRUS of the peripheral nerve in the forearm

**Figure 3:**
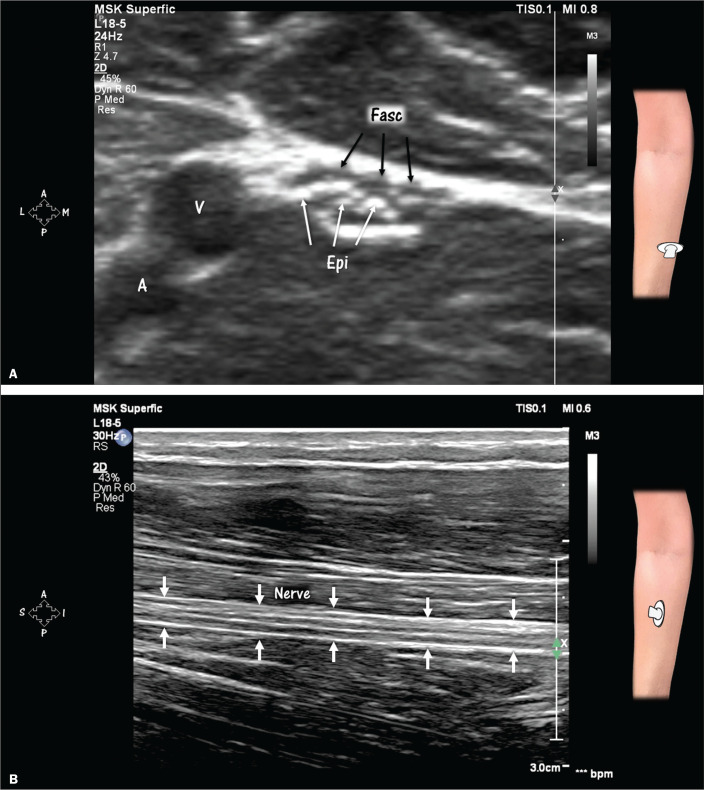
Peripheral nerve in the short and long axes on HRUS. A: Honeycomb appearance of a nerve in its short axis. Magnification, ×4.7. Compare the echogenicity of the nerve with that of the adjacent artery (A) and vein (V). The hypoechoic areas correspond to fascicles (Fasc, black arrows) and the hyperechoic areas correspond to the epineurium (Epi, white arrows). B: Nerve in its long axis (arrows). Note the alternating hypoechoic and hyperechoic lines and the “train-track” appearance. The hypoechoic lines correspond to fascicles, and the hyperechoic lines correspond to the epineurium.

To begin the HRUS examination of the peripheral nerve in the forearm, place the transducer at an easily identifiable anatomical landmark, such as the wrist or elbow, in the expected position for the nerve (Figure 1). Next, locate the nerve in the short axis, and then follow it up and down with the elevator technique^**(3,4)**^. In the short axis, the nerve has the characteristic honeycomb appearance, which facilitates its localization (Figure 3). In the long axis, the nerve is thin and elongated, with a mixture of hypoechoic and hyperechoic lines, with a “train-track” appearance^**(3,4,13)**^, as illustrated in Figure 3. In some places, it can be technically difficult to identify the peripheral nerve in the long axis because of its small dimensions, tortuosities along its course, and the edges of bone.

One factor that facilitates localization of the peripheral nerve is hyperechoic perineural fat, which is observed around the nerve in the intermuscular and fascial planes^**(4)**^. However, it can be difficult to demarcate the border between the epineurium and the perineural fat plane. If the nerve has poorly defined margins, measure the area by tracing the border around the most peripheral fascicles that have a hypoechoic aspect.

### Muscles and tendons

**Figure 4:**
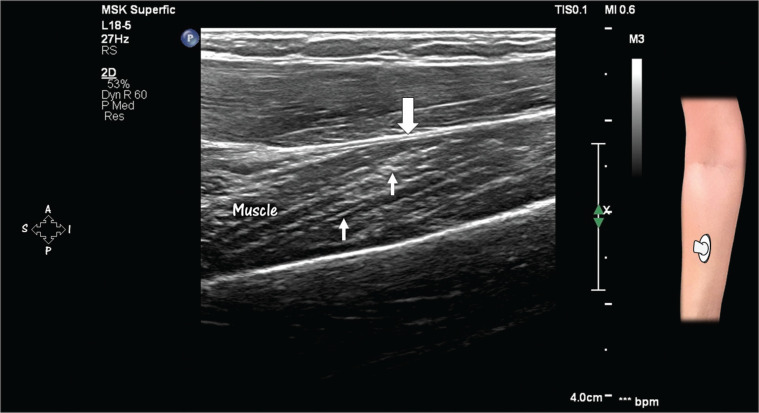
Striated aspect of a muscle in the long axis on HRUS. Note the thin bands of greater echogenicity within the muscle (arrows), corresponding to fibro-adipose supporting tissue. Note also the fascial envelope (wide arrow).

Typically, muscles are hypoechoic, are surrounded by fascia, and have thin internal echogenic septa that correspond to “fibro-adipose” supporting tissue^**(4,24)**^. Muscles have a striated or pennate appearance (Figure 4), an aspect that is more evident in the long axis^**(4,24)**^. Tendons appear more echogenic, compact, and fibrillar^**(4,23,25)**^. When followed proximally or distally, tendons end in muscle or bone^**(2)**^.

The morphological and structural parameters of the muscles evaluated with HRUS are trophism (thickness), echogenicity pattern, distribution of muscles involved by neuropathy, and fasciculations^**(25)**^. Changes in echogenicity in a muscle may be associated with edema, fatty degeneration, fibrosis, hematomas, calcifications, and masses. Edema and fatty degeneration can both increase the echogenicity of the muscle. Imaging findings should be correlated to the clinical history.

Tendinosis, which usually appears as tendon thickening and fibrillar pattern loss, is typically accompanied by hypoechoic changes^**(26)**^. Doppler may help in the detection of associated hyperemia. Partial and complete tears are characterized by areas of discontinuity of fibers, usually filled with fluid. Dynamic evaluation can facilitate the differential diagnosis between tendinosis and a torn tendon^**(26)**^.

### General guidelines for HRUS of forearm muscles

**Figure 5:**
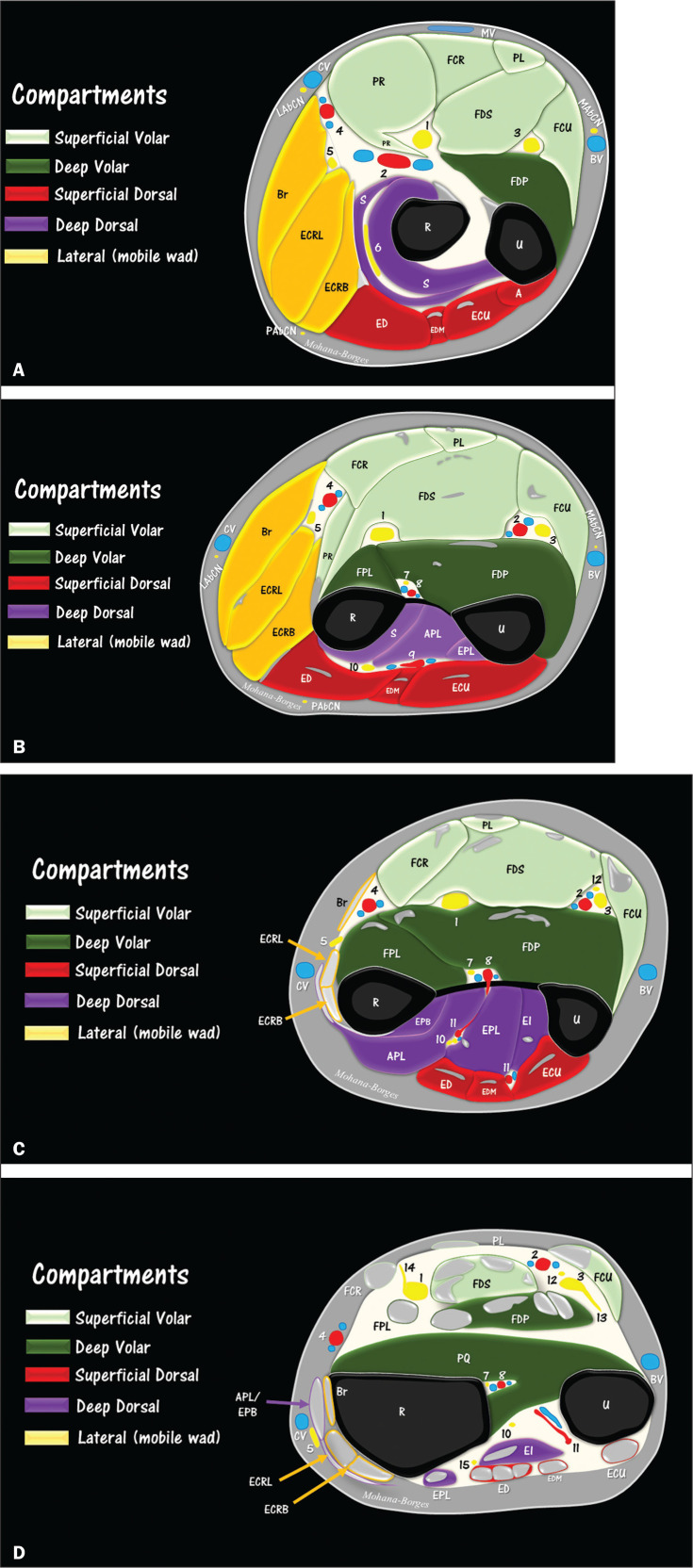
Diagrams of the forearm compartments. **A**: Level of the proximal forearm. Note the appearance of the ulnar head of the pronator teres (PR) muscle, between the median nerve (1) and the ulnar artery (2). The ulnar nerve (3) is located among the muscles flexor carpi ulnaris (FCU), flexor digitorum superficialis (FDS) and flexor digitorum profundus (FDP). At that point, the ulnar nerve is unaccompanied by vessels. At a slightly more proximal level, the ulnar nerve is accompanied by the posterior ulnar recurrent artery to the level of the cubital tunnel (not shown). Note the relative proximity between the radial artery (4) and the superficial branch of the radial nerve (5). The deep branch of the radial nerve (6) is located between the superficial and deep heads of the supinator muscle (S). At this proximal level, cutaneous nerves are better identified. Note the proximity of the lateral and medial antebrachial cutaneous nerves (LAbCN and MAbCN, respectively) in proximity to the cephalic vein (CV) and basilic vein (BV), respectively. FCR, flexor carpi radialis (muscle); PL, palmaris longus (muscle); Br, brachioradialis (muscle); ECRL, extensor carpi radialis longus (muscle); ECRB, extensor carpi radialis brevis (muscle); ED, extensor digitorum (muscle); EDM, extensor digiti minimi (muscle); ECU, extensor carpi ulnaris (muscle); MV, median vein of the forearm; R, radius; U, ulna; A, anconeus (muscle); PAbCN, posterior antebrachial cutaneous nerve. **B**: Level of the middle-proximal third of the forearm. Note the end of the supinator tunnel and the transition between the distal portion of the deep head of the supinator muscle (S) and the origin of the abductor pollicis longus (APL) muscle. Note the arch of the flexor digitorum superficialis (FDS) muscle over the median nerve (1). Note also the insertion of the pronator teres (PR) tendon in the lateral edge of the radius (R) and the origin of the flexor pollicis longus (FPL) muscle. Note the close relationship that the anterior interosseous nerve (7) has with the anterior interosseous artery and veins (8). The posterior interosseous artery (9) is close to the posterior interosseous nerve (10), which is the distal continuation of the deep branch of the radial nerve (6). Note also the close relationships that the ulnar nerve (3) and the superficial branch of the radial nerve (5) have with the ulnar (2) and radial (4) arteries and veins, respectively. EPL, extensor pollicis longus (muscle). **C**: Level of the middle-distal third of the forearm. Note the superficial branch of the radial nerve (5) piercing the fascia between the tendons of the brachioradialis (Br) and the extensor carpi radialis longus (ECRL). Note the divisions of the posterior interosseous artery (1) in the posterior compartment. From its origin, the palmar branch of the ulnar nerve (12) accompanies the ulnar nerve to the distal third of the forearm. EPB, extensor pollicis brevis (muscle); EI, extensor indicis (muscle). **D**: Level of the distal third of the forearm. Note the musculature of the pronator quadratus (PQ) and several tendons and myotendinous junctions crossing the region. Note the proximity of the superficial branch of the radial nerve (5) to the cephalic vein (CV). The origin of the dorsal cutaneous branch of the ulnar nerve (13) have a transverse course below the flexor carpi radialis (FCU) muscle. Also note the proximal portion of the palmar cutaneous branch of the median nerve (14) and the proximity to the fascia and tendon of the flexor carpi radialis (FCR). The posterior interosseous nerve (10) has several branches, one of the last identified at this level (15) innervating the extensor indicis (EI) muscle.

To begin the HRUS examination of the forearm muscles, place the transducer over the surface of the muscle in its short axial plane. Slide the transducer from one end to the other. Note the thickness, contour, and echogenicity of the muscle. Look for spontaneous wave muscle movements that may represent fasciculations, as well as for muscle projections related to fascial defects. Do the same analysis in the longitudinal plane.

To differentiate muscles and tendons from nerves, tilt the transducer and note the changes in the echotexture by anisotropy^**(22)**^. Tendons and muscles have greater anisotropy in relation to nerves and show more pronounced changes in echotexture related to the angle of insonation^**(23,26)**^.

Whenever possible, make panoramic scans of the muscles for an extended view of the area under study. Perform compression and decompression maneuvers, evaluating muscle consistency qualitatively or quantitatively by elastography. Evaluate tendons for signs of tendinosis, tears, and calcifications. Complement with Doppler to identify hyperemia.

## FOREARM COMPARTMENTS

### Division and function

There are different ways to divide the forearm into compartments. The following way is very useful for radiologists, surgeons, and anesthesiologists who need to understand the anatomy and correlate it with the function of each structure^**(9,10,27)**^. As depicted in Figure 5, the compartments are divided as follows^**(8-10)**^: volar (or flexor-pronator); dorsal (or extensor-supinator); and lateral (or mobile wad of Henry). The function of the volar compartment muscles is wrist flexion, digit flexion, and forearm pronation^**(27)**^. The function of the dorsal compartment muscles is wrist extension, digit extension, and forearm supination^**(27)**^. However, the lateral compartment muscles have a common fascial envelope that allows broader group sliding. That sliding is observed in movements of pronation and supination. This feature of the mobile wad allows the muscles to be retracted more easily during surgical procedures. The volar and dorsal compartments can also be subdivided into superficial and deep^**(28,29)**^, as detailed in Table 2. Some authors go further by subdividing the volar compartment into three layers, the flexor digitorum superficialis muscle being the intermediate layer^**(27)**^.

### Innervation of compartments

#### Volar compartment innervation

**Figure 6:**
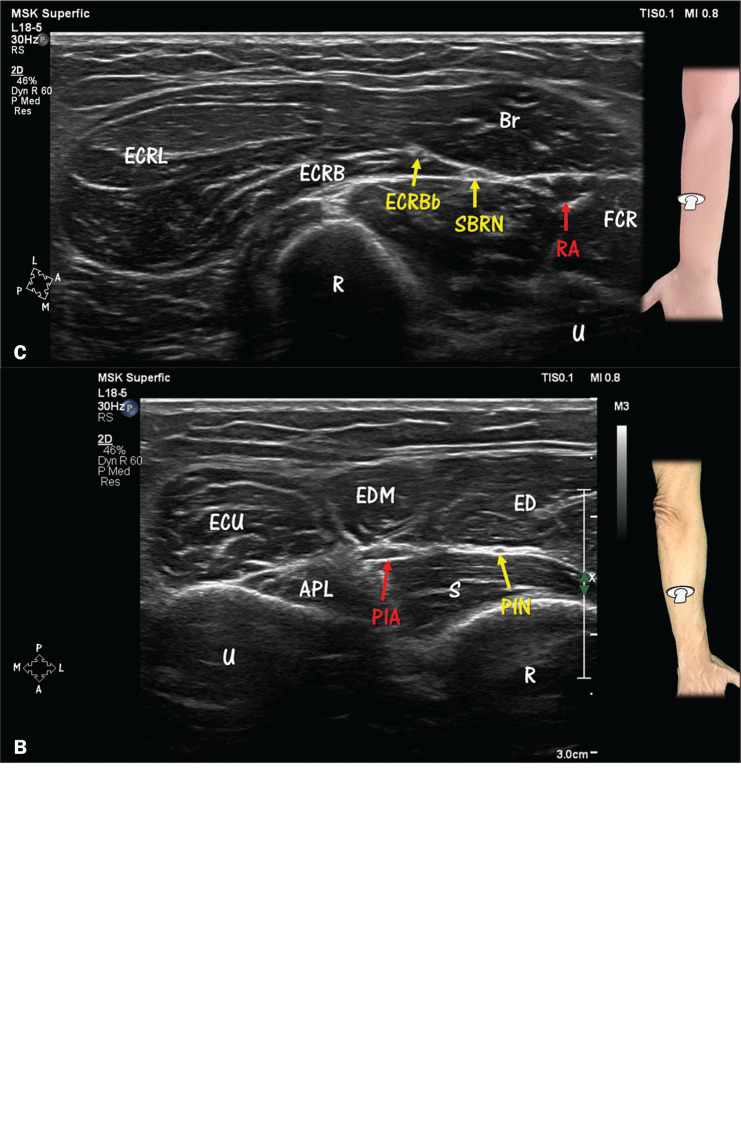
Forearm compartments and nerves. **A**: Volar compartment. Median nerve (MN) and ulnar nerve (UN) in the volar compartment of the forearm. Most of the muscles in this compartment are innervated by the median nerve, with the exceptions of the flexor carpi ulnaris (FCU) muscle and the medial part of the flexor digitorum profundus (FDP) muscle, both of which are innervated by the ulnar nerve. The ulnar artery (UA) can be seen close to the ulnar nerve. In the individual depicted, the radial artery (RA) is near the superficial branch of the radial nerve (SBRN) and the median nerve. PL, palmaris longus (muscle); Br, brachioradialis (muscle); FCR, flexor carpi radialis (muscle); FDS, flexor digitorum superficialis (muscle); ECRL, extensor carpi radialis longus (muscle); FCU, flexor carpi ulnaris (muscle); FPL, flexor pollicis longus (muscle); R, radius; U, ulna. **B**: Dorsal compartment. The compartment is innervated by the deep branch of the radial nerve and its continuation in the posterior interosseous nerve (PIN), with the exception of the anconeus muscle (not shown). Note the relative proximity of the PIN and the posterior interosseous artery (PIA) distal to the level of the distal arcade of the supinator muscle. EDM, extensor digiti minimi (muscle); ECU, extensor carpi ulnaris (muscle); ED, extensor digitorum (muscle); APL, abductor pollicis longus (muscle); S, supinator (muscle, deep head); U, ulna; R, radius. **C**: Lateral compartment. Among the three muscles of the compartment, two—the brachioradialis (Br) and extensor carpi radialis longus (ECRL)—are innervated directly by the radial nerve at the level of the arm. However, the extensor carpi radialis brevis (ECRB) muscle may be innervated by the deep branch of the radial nerve, by the superficial branch of the radial nerve (SBRN), or directly by the main trunk of the radial nerve. Note the superficial branch of the radial nerve coursing below the brachioradialis and close to the radial artery (RA). ECRBb, extensor carpi radialis brevis branch; FCR, flexor carpi radialis (muscle); R, radius; U, ulna.

As illustrated in Figures 1, 5, and 6, the median nerve and ulnar nerve innervate the musculature of the volar compartment^**(28)**^. Most of the muscles in this compartment are innervated by the median nerve, either directly or by its main forearm branch, the anterior interosseous nerve^**(30)**^. The medial corner of the compartment is innervated by the ulnar nerve^**(31)**^. The only forearm muscle innervated by the ulnar nerve is the flexor carpi ulnaris^**(28)**^. The flexor digitorum profundus has the peculiarity of double innervation, the medial part of the muscle being innervated by the ulnar nerve and the lateral part being innervated by the anterior interosseous nerve^**(32)**^. In the remaining musculature of the volar compartment, the median nerve innervates the muscles in the superficial layer and the anterior interosseous nerve innervates those in the deep layer^**(30)**^.

**Table 2:** Forearm compartments.

Compartment	Muscle	Innervation (nerve or nerve branch)	Arterial supply
Superficial volar	Pronator teres	Median	Anterior ulnar recurrent
	Flexor carpi radialis	Median	Radial
	Palmaris longus	Median	Anterior ulnar recurrent
	Flexor digitorum superficialis	Median	Ulnar
	Flexor carpi ulnaris	Ulnar	Posterior ulnar recurrent
Deep volar	Flexor digitorum profundus	Ulnar and anterior interosseous nerve	Anterior interosseous
	Flexor pollicis longus	Anterior interosseous nerve	Anterior interosseous
	Pronator quadratus	Anterior interosseous nerve	Anterior interosseous
Lateral (mobile wad of Henry)	Brachioradialis	Radial	Radial recurrent
	Extensor carpi radialis longus	Radial	Radial recurrent
	Extensor carpi radialis brevis	Radial, deep branch of the radial nerve, or superficial branch of the radial nerve	Radial recurrent
Superficial dorsal	Anconeus	Radial	Middle collateral/recurrent posterior interosseous
	Extensor carpi ulnaris	Posterior interosseous nerve	Posterior interosseous
	Extensor digiti minimi	Posterior interosseous nerve	Posterior interosseous
	Extensor digitorum communis	Posterior interosseous nerve	Posterior interosseous
Deep dorsal	Supinator	Deep branch of the radial nerve	Radial recurrent/posterior ulnar recurrent
	Abductor pollicis longus	Posterior interosseous nerve	Posterior interosseous
	Extensor pollicis brevis	Posterior interosseous nerve	Posterior interosseous
	Extensor pollicis longus	Posterior interosseous nerve	Posterior interosseous
	Extensor indicis	Posterior interosseous nerve	Posterior interosseous

#### Dorsal compartment innervation

The muscles of the dorsal compartment are innervated by the deep branch of the radial nerve and its continuation in the posterior interosseous nerve (Figures 1, 5, and 6), with the exception of the anconeus muscle^**(33-36)**^. The anconeus muscle is innervated directly by the radial nerve through the branch to the medial head of the triceps muscle.

Lateral compartment innervation

In the lateral compartment, the brachioradialis and extensor carpi radialis longus muscles are directly innervated by the radial nerve^**(34,35)**^. Although the extensor carpi radialis brevis muscle is innervated by the deep branch of the radial nerve in most people, there have been reports of other patterns, such as innervation directly by the main trunk of the nerve or by its superficial branch^**(35,37,38)**^, as can be seen in Figures 1, 5, and 6.

## CONCLUSION

For evaluating the peripheral nerves and muscles of the forearm, HRUS is an excellent method. Knowledge of the normal anatomy of the forearm improves the technical quality of the examinations, contributing to better diagnoses, as well as improving the safety and performance of interventional procedures.
